# Discrimination of primary and chronic cytomegalovirus infection based on humoral immune profiles in pregnancy

**DOI:** 10.1172/JCI180560

**Published:** 2024-10-15

**Authors:** Andrew P. Hederman, Christopher A.L. Remmel, Shilpee Sharma, Harini Natarajan, Joshua A. Weiner, Daniel Wrapp, Catherine Donner, Marie-Luce Delforge, Piera d’Angelo, Milena Furione, Chiara Fornara, Jason S. McLellan, Daniele Lilleri, Arnaud Marchant, Margaret E. Ackerman

**Affiliations:** 1 Thayer School of Engineering, Dartmouth College, Hanover, New Hampshire, USA.; 2 European Plotkin Institute for Vaccinology, Université libre de Bruxelles, Brussels, Belgium.; 3 Department of Microbiology and Immunology, Geisel School of Medicine, Hanover, New Hampshire, USA.; 4 Department of Molecular Biosciences, The University of Texas, Austin, Texas, USA.; 5 Université Libre de Bruxelles (ULB), Hôpital Universitaire de Bruxelles (H.U.B.), CUB Hôpital Erasme, Department of Obstetrics and Gynecology, Brussels, Belgium.; 6 ULB, H.U.B., CUB Hôpital Erasme, National Reference Center for Congenital Infections, Brussels, Belgium.; 7 Microbiology and Virology, Fondazione IRCCS Policlinico San Matteo, Pavia, Italy.

**Keywords:** Immunology, Infectious disease, Adaptive immunity, Antigen, Immunoglobulins

## Abstract

**BACKGROUND:**

Most humans have been infected with cytomegalovirus (CMV) by midlife without clinical signs of disease. However, in settings in which the immune system is undeveloped or compromised, the virus is not adequately controlled and consequently presents a major infectious cause of both congenital disease during pregnancy as well as opportunistic infection in children and adults. With clear evidence that risk to the fetus varies with gestational age at the time of primary maternal infection, further research on humoral responses to primary CMV infection during pregnancy is needed.

**METHODS:**

Here, systems serology tools were applied to characterize antibody responses to CMV infection in pregnant and nonpregnant women experiencing either primary or chronic infection.

**RESULTS:**

Whereas strikingly different antibody profiles were observed depending on infection status, limited differences were associated with pregnancy status. Beyond known differences in IgM responses used clinically for identification of primary infection, distinctions observed in IgA and FcγR-binding antibodies and among antigen specificities accurately predicted infection status. Machine learning was used to define the transition from primary to chronic states and predict time since infection with high accuracy. Humoral responses diverged over time in an antigen-specific manner, with IgG3 responses toward tegument decreasing over time as typical of viral infections, while those directed to pentamer and glycoprotein B were lower during acute and greatest during chronic infection.

**CONCLUSION:**

In sum, this work provides insights into the antibody response associated with CMV infection status in the context of pregnancy, revealing aspects of humoral immunity that have the potential to improve CMV diagnostics.

**FUNDING:**

CYMAF consortium and NIH NIAID.

## Abstract 2

Leveraging machine learning on antibody profiling datasets of primary and chronic CMV subjects allows for accurate prediction of infection status and new insights into the longitudinal antibody response.

## Graphical abstract

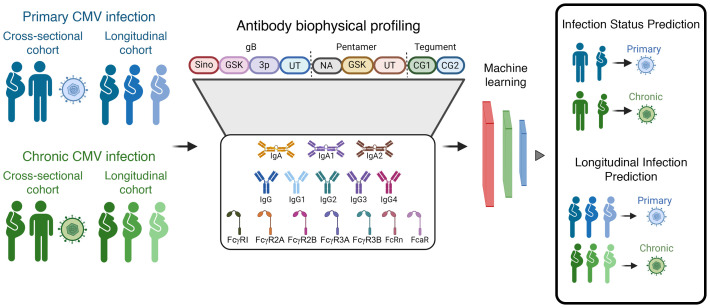

## Introduction

A member of the herpesvirus family, human cytomegalovirus (CMV) commonly manifests as a mild or asymptomatic infection. However, in individuals who are very young or immunocompromised, CMV can cause severe disease; it is the leading cause of congenital infection among newborns (1). In the United States alone it is estimated that 40,000 children are born with congenital CMV (cCMV) infection every year (1). This high burden is still likely an underestimate since many cases are asymptomatic at birth. Diagnosed infections are often severe, leading to an estimated 400 deaths and an additional 8,000 cases presenting with permanent disabilities, including speech and language impairment, hearing loss, mental disability, cerebral palsy, and vision impairment, annually (1, 2). Fetal infection results from intrauterine transmission and is most likely to occur when a mother experiences primary CMV infection during pregnancy (3–5). The difference in fetal infection risk between primary and reactivated maternal CMV infection is striking, with approximately one-third of primary infections leading to CMV infection of the fetus compared with under 3.5% estimated to result from CMV reactivation or superinfection (6–10). New insights into CMV infection during pregnancy that could contribute to identification of pregnancies at greatest risk, efficient testing of new therapeutic interventions, and vaccines that could modify transmission risk are urgently needed (11).

Pregnant women present a population that is not immunodeficient, but in a unique state of immune regulation in order to ensure fetal tolerance, and can transmit CMV to their fetus during pregnancy. While the differing risk of transmission associated with maternal seropositivity provides strong evidence that preconception immunity plays a protective role, studies evaluating the clinical potential of CMV-hyperimmune globulin to improve neonatal outcomes have yielded mixed results (12–14). While possible contributing factors to these results include low potency and insufficient serum persistence of the CMV-hyperimmune globulin, more data is needed to better understand the clinical potential of this intervention in the context of vertical transmission (15, 16). It has been speculated that these differing outcomes may relate to gestational age and the timing of the intervention following maternal infection and therefore be impacted by both the reliability of the diagnostic approach and strictness in the definition of primary infection cases. Because the suitability of these approaches for application during pregnancy can be supported by comparison of responses in individuals who are pregnant and nonpregnant during primary infection, developing a deeper understanding of humoral immune profiles in this unique immune state may have implications for the clinical development of diverse small molecule and biologic antiviral interventions.

Short of these goals, given the widely diverging congenital infection risks associated with primary and chronic infection, confident discrimination between these states is crucial for identifying newborns with highest risk of cCMV infection. Complementing virological assessment, discrimination of primary and nonprimary infection using serology may allow antiviral treatment in pregnant women and hearing and learning interventions in newborns to be initiated early (17, 18). Current diagnostic assays for pregnant women are performed using serology measurements for IgM and IgG avidity, which decrease and increase over time following infection, respectively, while a PCR-based urine test, often following a saliva PCR screening test, is the standard for newborns (17, 19–21). However, although CMV is a common infection, there is no universal standard diagnostic assay across countries and healthcare centers (22), introducing variability in care and posing challenges to clinical trial design for testing of novel interventions. Additionally, many countries, including the United States, do not routinely screen for CMV in pregnant women, whereas some European countries as well as some individual states (e.g., Minnesota) do perform routine screening — adding another layer of complexity to clinical data regarding the impact of maternal CMV status, antibody responses, and timing of infection as assessed by differing diagnostic measures and across populations. Irrespective of these details, following a primary CMV diagnosis during pregnancy, clinicians typically counsel pregnant women about the future risks to their fetus.

The dichotomy in fetal risk profiles of primary and nonprimary CMV infection has important implications for predicting transmission risk for pregnant women. Beyond this classification, the specific timing of CMV infection is also a crucial factor in the potential of severe congenital disease, with a primary infection in the first term of pregnancy leading to higher likelihood of severe cCMV infections than a primary infection in the second or third trimester (23). Further insights into relative risk profiles are challenged by imperfect precision in defining and estimating the timing of primary infection, particularly during pregnancy, which has been associated with varying and dynamic immunological state changes (24), including differences in humoral responses to both infection and vaccination that vary with gestational age and affect response magnitude, kinetics, and character (25, 26) in ways that have the potential to impact CMV diagnoses based on serological testing. As a result, more accurate and confident diagnosis of primary and nonprimary CMV infection, specifically in the context of pregnancy, would benefit the medical community and affected birthing parents by providing clearer and more definitive insights into their CMV infection status and more optimal support studies of associated risk to the fetus.

Previous work in a variety of infectious disease settings has shown that antibody responses evolve over time, exhibiting complex patterns in response magnitude and characteristics (27–29), and that they can also differ in association with pregnancy (29–31). Pregnant women with primary CMV infection represent a unique intersection of these complex antibody response scenarios with established clinical significance. Here, leveraging carefully curated cohorts, machine learning, and highly multiplexed assays to capture a wide range of antibody response attributes over time, we evaluate how responses to primary or nonprimary CMV infection vary over time and in association with pregnancy. In doing so, this work defines the limited role pregnancy plays in modifying humoral responses to CMV infection and identifies the unusual kinetic profile of IgG3 responses to different viral antigens as a potentially new means of identifying and dating the onset of primary infection, two factors relevant to clinical studies evaluating and reducing risk of cCMV infection for affected pregnancies.

## Results

### Antibody profiles distinguish primary from chronic CMV infection.

**Figure 1:**
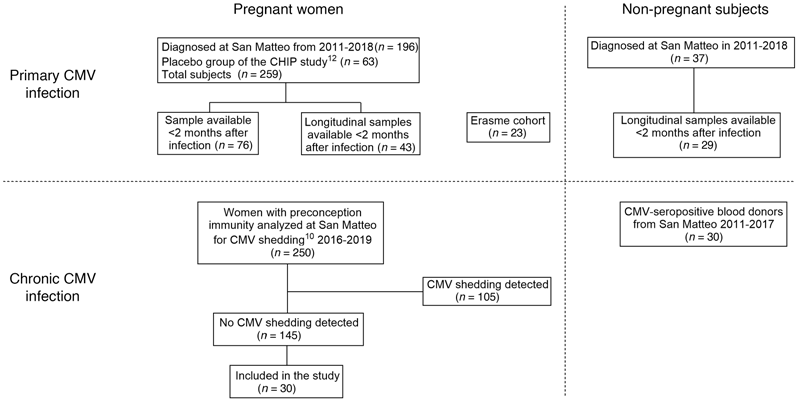
Diagram of study cohort participants.

Antibody responses were profiled among individuals who were pregnant and not pregnant with either primary or chronic CMV infection (Figure 1 and Table 1), with primary infection strictly defined by CMV-specific IgG seroconversion, CMV-specific IgM antibody detection, low IgG avidity index, and/or CMV DNAemia. Conversely, chronic infection was defined by seropositivity in the absence of these diagnostic measures. Antibody responses following primary or chronic CMV infection were profiled in cross sectional and longitudinal cohorts. (Supplemental Figure 1 and Supplemental Table 1; supplemental material available online with this article; https://doi.org/10.1172/JCI180560DS1). Specifically, a cross-sectional cohort included individuals who were grouped as pregnant primary (n=74), nonpregnant primary (n=27), and nonpregnant chronic (n=40). Additionally, a set of longitudinal samples were available from participants who were pregnant and presented with either primary (n=57) or chronic (n=36) CMV. To more comprehensively understand how antibody profiles vary among individuals with CMV infections, in addition to participants who were CMV naive (n=9), antibodies specific for CMV tegument protein (32), glycoprotein B (gB), and the pentamer complex were characterized for isotype, subclass, and Fc Receptor (FcR) binding capacity (Supplemental Figure 1 and Supplemental Table 1). We profiled the antibody response, examining a diverse set of antibody features and CMV antigens, including gB and pentamer complexes from several different sources, with the aim of gaining a more complete view of the humoral immune response to primary or chronic CMV infection. Glycoproteins tested included several that are commercially available (Sino, Sino Biologicals; and NA, Native Antigen), as well as others that have been characterized in structural studies conducted with an eye toward vaccine development (GSK, Glaxo-Smith-Kline) (33, 34), and by academic groups (UT, University of Texas, Austin, Texas, USA) (35, 36), including a modified form of gB with mutations intended to reduce aggregation and eliminate the furin cleavage site (UT gB) and a form to favor the prefusion conformation by including engineered proline mutations (3p gB). Tegument antigens included portions of pp150 (UL32) and pp52 (UL44) proteins, including CG1 (pp150/2-pp52/3, comprised of amino acids 495–691 and 862–1048 of pp150) and CG2 (pp150/7-pp150/1, comprised of amino acids 695–864 of pp150 and 297–433 of pp52), which were previously reported to be immunodominant targets for sero diagnosis of primary and chronic CMV infection (37, 38).

**Figure 2:**
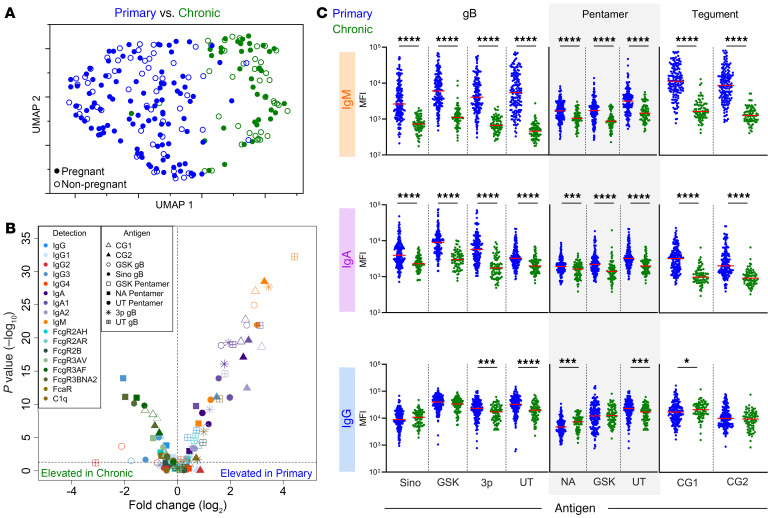
Antibody features distinguish primary from chronic CMV infection but not pregnancy status. (**A**) Uniform manifold approximation (UMAP) biplot of antibody features excluding IgM. Distinct clusters of participants with primary (blue, *n* = 158) and chronic (green, *n* = 76) infection but not pregnancy status (hollow and filled symbols) are observed. (**B**) Volcano plot of each CMV-specific antibody feature assessed. Volcano plot represents the log_2_ fold change (x-axis) against the –log_10_ *P* value (Mann-Whitney test: *P < 0.05, **P < 0.01, ***P < 0.001, and ****P < 0.0001). Antibody specificities (antigen) are indicated by shape and Fc characteristics (detection) indicated by color. (**C**) IgM, IgA, and IgG binding to CMV antigens (further described in Supplemental Table 1). Data are the mean median fluorescent intensity (MFI) values of technical replicates. Solid red line indicates median.

To define differences in antibody profiles among participant groups that go beyond the measures typically used in clinical diagnosis, we performed UMAP analysis on CMV-specific antibody features after specifically excluding IgM (Figure 2A). This unsupervised analysis revealed that the main aspects of differentiation among participants related to infection status; striking differences in the antibody response were observed between individuals with primary as opposed to chronic infection. In contrast, limited differences in the global response profiles were observed between individuals who were pregnant and not pregnant. While univariate analysis between pregnant and nonpregnant female participants during primary infection did reveal some nominally statistically significant differences (Supplemental Figure 2), we could not exclude the possibility that these differences resulted from a difference in timing of diagnosis or were impacted by differences in age between cohorts. The former may be likely, as similarly subtle differences were observed between primary participants in these groups and an independent cohort (Erasme Hospital) of pregnant women with primary CMV infection (*n*=23), as defined using different assays and criteria by a different medical center, and whose blood was collected over a broader range of days after diagnosis (39) (Supplemental Figure 3). We additionally explored whether biological sex impacted antibody responses, as some cohorts included participants who were male and female, but found limited differences (Supplemental Figure 4). Given these observations and the lack of robust differences associated with pregnancy status or biological sex, these variables were not considered in subsequent analyses.

We next investigated which individual features of the immune response might contribute to clustering of participants in primary and chronic infection groups. Visualizing the degree and confidence of differences in antibody response features between primary and chronic infection status groups revealed aspects of the humoral response that consistently differed (Figure 2, B and C). Distinct levels of CMV-specific IgA, IgM, and FcR binding antibodies were observed between primary and chronic infection groups across multiple CMV antigens. IgM responses were elevated across all antigens for the primary infection group, which was expected as IgM titers were used to define infection status. Interestingly, total CMV-specific IgA and both IgA1 and IgA2 subclasses were elevated in participants with primary infection. Somewhat surprisingly, total IgG had very modest differences between primary and chronic infection groups.

Given the striking differences among groups, we next wanted to further examine individual responses for IgM, IgA, and IgG across the panel of CMV antigens. As expected, IgM responses were strongly and significantly elevated among participants with primary infection, as were IgA responses across diverse antigen specificities (Figure 2C). Interestingly, and in contrast with total IgG response levels (Figure 2C), which exhibited statistically significant but relatively small differences in response magnitude, FcγR-binding antibodies were both significantly and strongly elevated among participants with chronic CMV infection (Figure 2B and Supplemental Figure 5), suggesting the presence of qualitative differences in the antibodies present during primary and chronic infection that may affect the effector functions mediated by this class of widely expressed innate immune cell receptors.

### IgG subclasses and CMV antigens display distinct profiles in primary and chronic infection.

**Figure 3:**
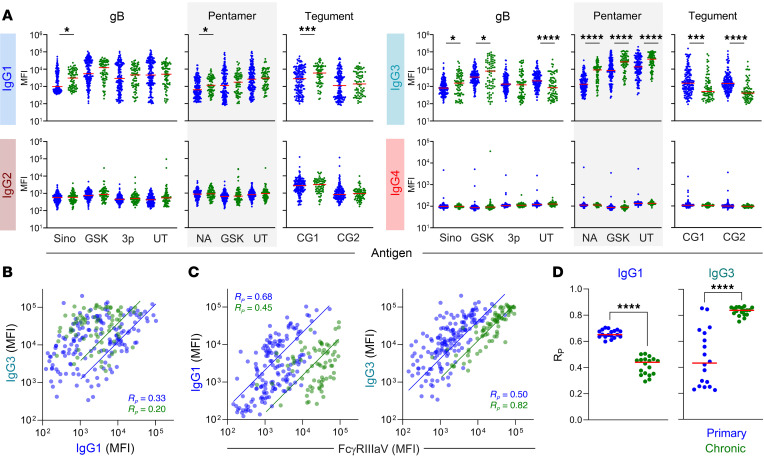
IgG subclasses display distinct profiles in primary and chronic infection depending on antigen specificity. (**A**) Levels of gB-, pentamer-, and tegument-specific IgG1, IgG2, IgG3, and IgG4 antibodies in individuals with primary (blue, *n* = 158) or chronic (green, *n* = 76) CMV infection. (**B**) Scatterplot and fit lines of levels of (UT) pentamer-specific IgG1 and IgG3 responses by infection status. Pearson correlation coefficient (R_P_) indicated in inset. (**C**) Scatterplot and fit line of levels of (UT) pentamer-specific IgG1 (left) and IgG3 (right) versus (UT) pentamer-specific FcgRIIIaV-binding antibodies. Pearson correlation coefficient (R_P_) indicated in inset. (**D**) Pearson correlation coefficients (R_P_) for each pentamer antigen tested across FcgR by infection status. Solid lines denote group medians; differences between groups were assessed by Mann-Whitney test (*P < 0.05, **P < 0.01, ***P < 0.001, and ****P <0.0001); values presented are median fluorescent intensities (MFI).

The observation that FcγR binding antibodies but not total IgG were reliably elevated in participants with chronic CMV was intriguing, and suggested that differences in induction of IgG subclasses, which differ dramatically in their FcR binding capacity, may exist. However, with the exception of IgG2 responses to tegument, IgG2 and IgG4 responses to CMV antigens were uncommon, and differences in the levels of these more functionally inert subclasses were not observed. Humoral responses to viral infections are generally dominated by IgG1 and IgG3 antibodies, which both bind well to activating FcγR and have the potential to elicit the potent antiviral activities of the complement cascade and innate immune effector cells (40, 41). While statistically significant differences were observed for a subset of antigens tested, IgG1 responses, which are typically dominant following acute viral infections (42, 43), were generally similar in primary and chronic infection (Figure 3A). Only a single of the tested gB, pentamer, and tegument proteins tested showed a statistically significant difference, and each of these demonstrated only a small increase among the chronic infection group. In contrast, IgG3 responses were more uniformly and strikingly distinct between groups. Perhaps most surprisingly, the direction of these differences varied by antigen specificity. Antibody responses to tegument proteins exhibited elevated levels in primary infection, but elevated levels of gB and pentamer-specific IgG3 were observed among the chronic infection group (Figure 3A). While all pentamer complexes tested showed this profile, responses to recombinant gB proteins were more variable, with 2 gB preparations showing elevated levels among participants with chronic CMV, and a single preparation showing elevated levels in primary infection. While some distinctions in IgG subclass composition in the context of primary and latent herpesvirus infections have been reported (44–48), how well they may support discrimination between primary and chronic CMV infection is not known. In sum, while IgG1 responses tended to either persist or increase between primary and latent infection classes, IgG3 responses differed dramatically by antigen specificity; responses to tegument were higher among participants with primary infection, but responses to gB and pentamer were instead elevated in chronic infection.

While UT pentamer-specific IgG1 and IgG3 levels were correlated with each other among participants with primary infection (R_P_=0.33, *P* < 0.0001), they were not well correlated among individuals with chronic infection (R_P_=0.20, *P* =0.08) (Figure 3B). This difference in degree of correlation between IgG1 and IgG3 responses between infection status states was consistent across pentamer proteins evaluated. Additionally, the strength of correlation observed between these IgG subclasses and the FcγRIIIaV-binding capacity of pentamer-specific antibodies changed over time, with stronger correlations between IgG1 and FcγRIIIaV observed in primary infection, but IgG3 and FcγRIIIaV binding in chronic infection (Figure 3C). These patterns were consistent across FcγRs and pentamer proteins tested (Figure 3D), suggesting that the pool of antibodies most capable of eliciting FcγR-dependent effector functions changes in composition over the course of infection. These temporal differences may have important implications to both viral pathogenesis based on the potential influence of IgG subclass on immune evasion mediated by viral Fc receptors such as gp34, gp68, gp95, and gpRL13 (49–52), as well as to host defense based on their differing capacities to induce robust antibody effector functions directed against free virions or virally infected cells.

### Prediction of CMV infection status using machine learning.

**Figure 4:**
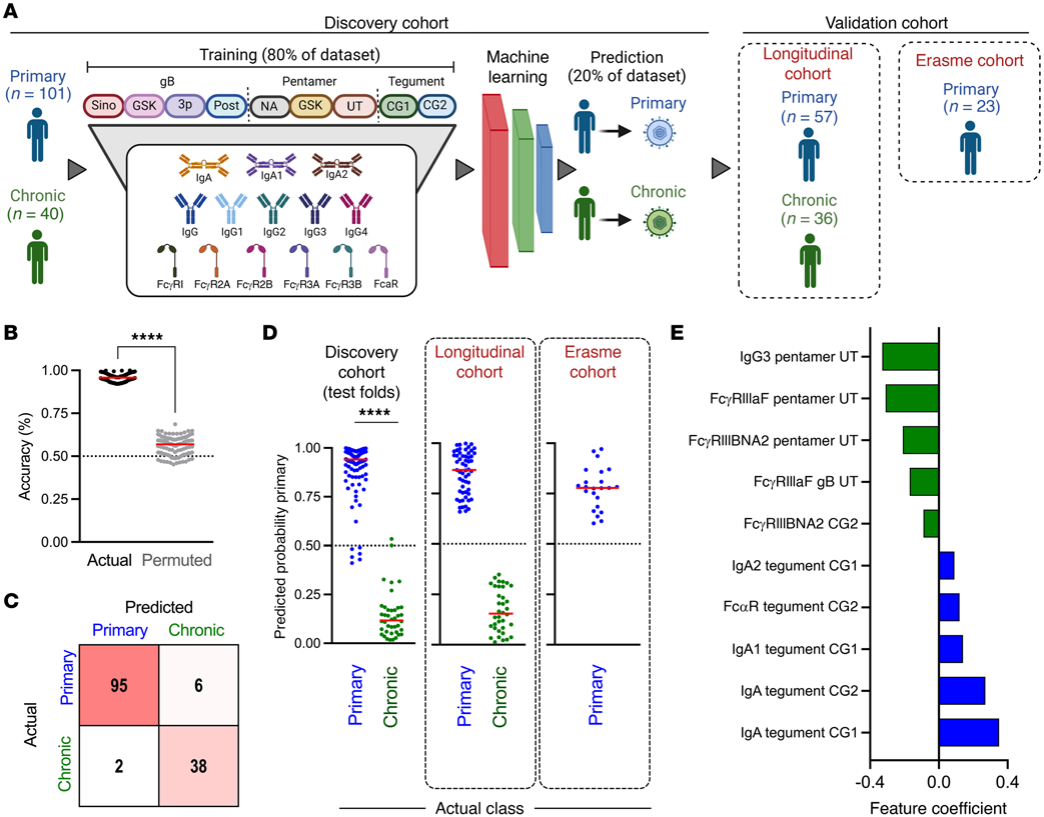
Machine learning accurately predicts primary or chronic CMV infection status. (**A**) Schematic overview of cross-validated machine learning workflow employing antibody profiling data to discriminate between primary and chronic infection status in discovery and validation cohorts. (**B**) Prediction accuracy for 100 repeated 5-fold cross-validation runs on actual (black) and permuted (gray) class labels. (**C**) Confusion matrix of predicted versus actual class labels in the median model for 5-fold cross validation. (**D**) Class probabilities of each sample in the discovery set when evaluated as a test sample in the cross-validation run exhibiting median performance (left) and for the cross-sectional (center) and Erasme (right) validation cohorts using the final model. (**E**) The identities and coefficients of the features making the largest positive (*n* = 5) or negative (*n* = 5) contributions to the final model. Solid lines denote group medians; differences between groups or conditions were assessed by Mann-Whitney test (*P < 0.05, **P < 0.01, ***P < 0.001, and ****P <0.0001).

Next, because unsupervised analysis of antibody profiles revealed clear distinctions between primary and chronic CMV infection, we applied supervised machine learning to explore the ability of a model to accurately discriminate primary and chronic CMV infection and to identify features that contribute to class differentiation (Figure 4A). Based on its simplicity and interpretability, we employed a logistic regression framework with regularization to classify primary or chronic CMV infection based on antibody profiling data while reducing the risk of overfitting associated with high-dimensional data. Again, IgM responses were excluded because they were used in clinical class assignment of the study groups. The model was trained on 80% of profiles for participants with primary and chronic infection while the remaining 20% was used for testing. Further, repeated 5-fold cross validation was employed so each participant would be part of the test set and representative accuracy across different folds could be defined.

Model predictions were highly accurate; across 100 repeated 5-fold cross validation runs, the median model accuracy for predicting primary or chronic CMV infection was 94% (Figure 4B). In contrast, prediction results were essentially random when training and testing was performed after permutation of class labels, which serves as a means to assess model robustness by measuring the potential for overfitting. Misclassifications were not biased toward one or the other class, as shown for the cross validation run repeat presenting median accuracy as a representative confusion matrix (Figure 4C). Classification calls for actual but not permuted class labels were both typically correct and assigned to respective classes with high probability (Figure 4D). The relatively few incorrect classifications were typically close to the decision boundary. Given this evidence of model accuracy and robustness, a final model was trained on all participants in the discovery cohort. When applied to an independent longitudinal set of 57 primary infection and 36 chronic infection samples serving as a validation cohort, this model resulted in similarly high confidence and perfect classification accuracy (Figure 4D). Additionally, an independent sample set of primary infection cases from a distinct medical center (Erasme) also yielded excellent accuracy (Figure 4D). Given this validation, the top features employed in the final model were examined (Figure 4E). The features with largest positive coefficients, which serve to identify primary participants, were primarily IgA-related antibody responses directed to tegument antigens (4 of 5 features). Conversely, features with large negative coefficients, useful to identify chronic infection, were typically related to IgG3 and FcγRIII-binding capacity of response to gB and pentamer (4 of 5 features). Collectively, these modeling results point to specific antibody response attributes, distinct from traditionally considered parameters such as IgM and IgG avidity, as being excellent candidate markers for distinguishing primary and chronic infection status. Among these, tegument-specific IgA and glycoprotein-specific IgG3 responses stand out as robust contributors that could be easily evaluated.

### Modeling longitudinal responses to CMV infection reveals a molecular clock of antibody responses.

**Figure 5:**
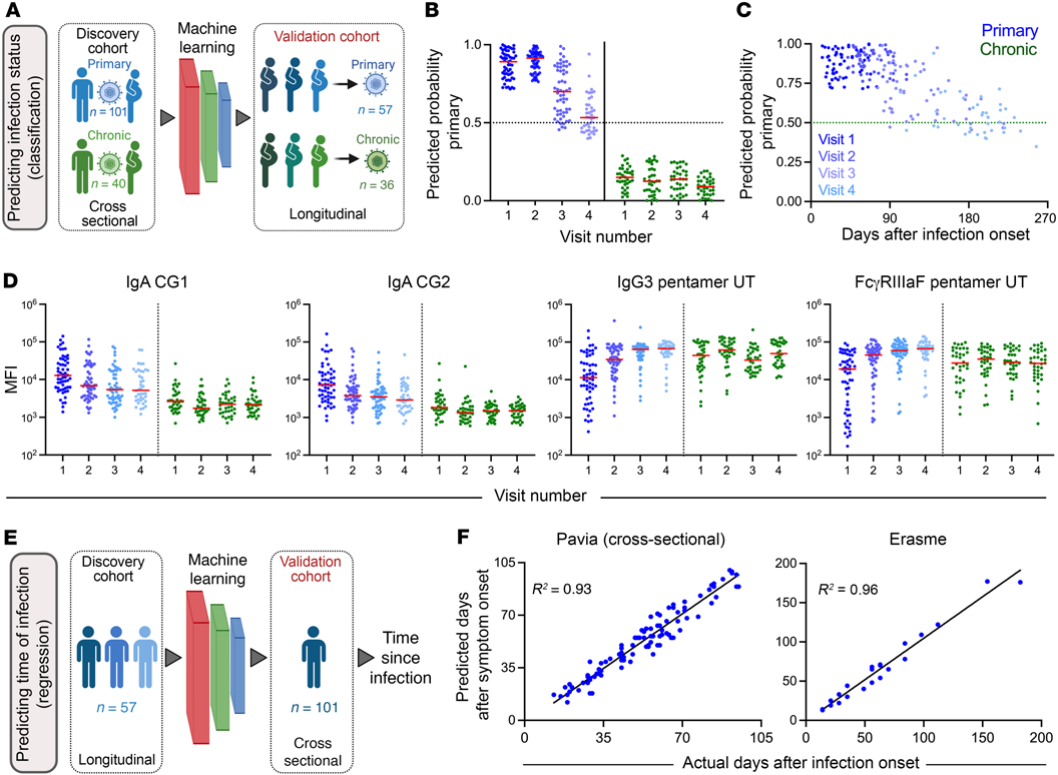
Longitudinal models define a molecular clock of CMV primary infection. (**A**) Analysis overview. The infection status classification model trained on the cross-sectional cohort was applied to longitudinal samples available from the validation cohort. (**B**) Class probabilities of each sample in the longitudinal cohort over sample collection visits for individuals with primary (blue) and chronic (green) infection. (**C**) Scatterplot of class probabilities for participants defined as having primary infection at visit 1 over time. (**D**) Scatterplots of features employed by classification model to predict infection status over time in the longitudinal cohort. (**E**) Analysis overview. Primary infection samples from the longitudinal cohort samples were used to train a regression model to predict time since infection (days after symptom onset) that was applied to the primary samples from the cross-sectional cohort. (**F**) Scatterplot of model predictions of time since infection when primary samples used for predicting days after symptom onset. The cross-sectional Pavia (left) and Erasme (right, *n* = 23) samples w0ere used as distinct validation cohorts. Data shows the measure of the predicted label and its closeness to the true label.

The excellent discrimination of primary and chronic CMV infection in the validation cohort led us to next explore how class predictions related to longitudinal development of humoral immune responses to infection. To this end, subsequent samples were available for the validation cohort over a series of up to 4 visits that extended out to half a year after initial sampling or infection onset. We explored the longitudinal cohorts in unsupervised fashion across all features by projecting serial time points on a UMAP model developed on visit 1 samples (Supplemental Figure 6). Again, participants with chronic CMV clustered distinctly from participants with primary infection. Strikingly, subsequent samples from the primary samples shifted closer to the chronic samples, consistent with the existence of humoral response characteristics that exhibit consistent changes over time following primary CMV infection. Next, the model trained on the initial cohort was used to predict class for longitudinal samples from the validation cohort (Figure 5A). Whereas participants with chronic infection were consistently classified as such with similarly high confidence at all subsequent visits, participants with primary infection at the initial time point became less confidently classified as primary at subsequent visits (Figure 5B). By visit 4, the majority of samples from participants with primary infection at their initial visit exhibited class probabilities below the lowest observed at the initial sampling. However, because visits were not consistently spaced in time between participants, these longitudinal profiles can be more meaningfully compared over time after symptom onset (Figure 5C). Despite the imperfect reliability of projected timing of infection, the classification model probabilities presented a clear relationship with time. Samples fell below the midpoint on the classification scale as early as 90 days after infection onset, but failed to reach values typically observed among individuals with chronic infection even out at 250 days after infection, suggesting a relatively prolonged transition to reaching the chronic state profile. As expected, the individual antibody response features making the greatest contributions to the classification model also demonstrated clear changes among participants with primary, but not chronic, infection over time (Figure 5D).

To this point, machine learning models have only been concerned with making predictions on the probability of a sample belonging to either the primary or chronic infection class. However, given the clear ability for these binary classification models to provide insight into time since infection, we next sought to evaluate models explicitly trained for this specific purpose. For this purpose, longitudinal profiles of the primary infection cases across visits were used to train a model to predict time since symptom onset as a continuous variable (Figure 5E). The resulting linear regression model, which showed good robustness in the context of 5-fold cross-validation (Supplemental Figure 7A), was then used to predict days after infection for the cross-sectional primary samples. This model, which relied primarily on IgG3 features (Supplemental Figure 7B) showing strong time dependence (Supplemental Figure 7C), was then applied to the cross-sectional cohort. Predictions of time since symptom onset in the unseen validation cohort exhibited excellent accuracy for both this and the independent Erasme cohort (Figure 5F). Overall, this analysis demonstrated that antibody profiles in individuals with CMV infection exhibit generalizable temporal patterns in their dynamic antibody responses during primary infection that can be used to retrospectively date the time of infection.

## Discussion

Presently, discrimination of primary and nonprimary CMV infection in the context of pregnancy is used in counseling regarding the risk of cCMV infection based on the lower risk of congenital infection associated with nonprimary infection and the prescription of antiviral therapies. Beyond this value, in the absence of an effective vaccine, a deeper understanding of how immune responses differ in association with infection history and transmission risk has the potential to contribute to the development of new interventions. Here, high dimensional antibody profiles beyond IgM levels and IgG avidity were developed from a commonly used multiplex assay format and supervised and unsupervised machine learning was used to differentiate primary and chronic infection and pregnancy status.

Whereas limited or no differences in humoral responses were associated with pregnancy status, our study showed clear distinctions in antibody profiles between primary and chronic infection cases. Both composites profiles and individual antibody features related to antigen-specificity and immunoglobulin isotype, subclass, and binding to Fc receptors demonstrated these distinctions. Importantly, these differences extended beyond those previously known to exist and which are presently applied to support clinical diagnosis. While further work is needed to assess the performance of a restricted set of the features identified as useful differentiators in this study for routine clinical laboratory use, IgG3 responses to pentamer, and to a lesser extent gB, along with IgA responses to tegument proteins were identified as good potential candidates. Whereas multiplex assays were employed here, we expect that assessment of these responses could be readily adapted to other assay formats commonly used in clinical testing labs. The number and longitudinal profiles of the features that distinguish infection history suggested that a sort of humoral clock could be defined in order to time the onset of primary CMV infection. Indeed, supervised machine learning models that reliably captured how antibody responses to CMV infection varied over time were learned and validated on independent samples.

Beyond potential clinical utility, the defining features of how responses to primary CMV infection transition over time is relevant to understanding the evolution of the immune response to this member of the notoriously immune-evasive Herpesviridae family (53, 54). Current diagnostic methods rely on detection and levels of IgM and of IgG avidity. The presence of IgM alone is insufficient to diagnose a primary CMV infection; poor correlation between commercial tests have been reported (55) and false positive primary status calls can result from both persistence of IgM as well as boosting in response to reactivation (56, 57). Likewise, a positive test for CMV-specific IgG indicates that an individual is positive for CMV but provides little information into the time since infection (17, 58). However, the combination of IgM positivity and low IgG avidity is generally considered to be a reliable indicator of primary CMV infection; though interpretation of IgG avidity tests is confounded by low levels of CMV-specific IgG, intermediate responses raise classification issues, and these indicators are supported by clinical studies of small size. Coupled with the lack of an international standard serum panel of samples from primary infection, the difficulty inherent to establishing a uniform, accurate, and robust diagnostic method is clear (59). Our data demonstrate that there are other changes in the secreted antibody repertoire that reliably occur over consistent time periods during primary infection and which could provide clinical utility, enhancing confidence in enrolment of participants in both interventional and observational studies.

Prior studies of IgG subclasses of CMV-specific antibodies have consistently reported the IgG1 and IgG3 bias typical of antiviral antibodies but noted that subclass responses and total IgG titers can be discordant (45, 47). Further, they have attributed relatively greater neutralization potency to this minor portion to IgG3 (46). The greater sensitivity of multiplexed assays over classical Western blots, as well as their ability to define responses directed toward specific antigens compared with whole virus or infected cell lysate offered the possibility to define previously unappreciated aspects of how the humoral response to CMV infection changes over time. To that end, we were surprised to observe increasing levels of IgG3 specific for viral glycoproteins over time. Whereas IgG3 responses are usually associated with acute infections and typically wane over time (43, 60–62), they were observed to increase across both cross-sectional and longitudinal cohorts in an antigen-specific manner. Namely, whereas IgG3 responses to tegument proteins showed the typical pattern and decreased while IgG1 responses were stable or increased, IgG3 responses to pentamer increased while IgG1 responses remained largely stable over time. Intriguingly, memory B cells (MBC) specific for gB are known to exhibit phenotypic states distinct from those specific for tegument antigens (63). The reduced frequency of MBCs with effector potential specific for glycoprotein compared with tegument observed in this prior study may relate to the altered kinetics of IgG subclasses observed here. While the mechanistic underpinning and the biological implications of this unusual pattern have yet to be determined, as noted previously, CMV expresses multiple Fc binding proteins, including gp34, gp68, gp95, and gpRL13, some of which are known to antagonize host FcγR (64). Unlike the viral FcR of HSV, these proteins have been reported to bind to all human IgG subclasses (49, 65), though affinities and potential differences among the IgG allotypes have not been reported. Additionally, how these 2 surface proteins contribute to viral pathogenesis is incompletely understood (66).

Limitations of this study include the fact that the majority of samples were sourced from a single geographic region, leaving open the possibility that differences associated with host genetics and environmental and infectious disease exposure history affect these observations. Additionally, the onset of infection could only be estimated rather than defined with precision, which confounds models of this parameter and points to the value of further evaluation in the context of infections for which definitive timing is known in order to provide greater confidence. While sex as a biological factor was investigated, cohorts were skewed toward female representation and had limited power to define sex-based differences. Further, the panel of antigens tested was not exhaustive; for example, no viral FcR were included, and while responses to common vaccine antigens were evaluated, surface glycoprotein coverage was not comprehensive. Among tested antigens, reactivity patterns sometimes differed in association with different sequences, conformational states, presence or absence of glycosylation sites, recombinant protein expression host cell lines, and other factors, the contributions and importance of which have yet to be defined. These and other factors may represent worthwhile directions for future study.

Lastly, while relationships between maternal immune responses and transmission of cCMV are of exceptionally high clinical relevance to both risk management and vaccine research and development, this study focused on CMV infection history. However, while risk is considerably greater during primary infection, there are certainly differences in virologic, innate immune, and other factors that contribute to these differing risk profiles (67–70). The influence of maternal antibody responses is unclear, particularly given the conflicting results in studies of passive antibody therapy in the context of primary maternal infection (71, 72). While the dosing and frequency of hyperimmune globulin also differed in these studies, a positive effect was only observed in women with very early infection, pointing to the potential importance of accuracy in the dating of infection recency. Recent studies have started to explore the role that antibody responses play in CMV transmission in a rhesus macaque model (73, 74). However, data in humans is limited and often confounded by the differing risk of infection associated with primary compared with nonprimary infection (75). Indeed, our observations illustrate the extent of this confounding and point to the value of highly curated cohorts targeted to address the critical unmet need for a CMV vaccine. Study within primary and chronic infection groups will be required to unambiguously relate risk of transmission with attributes of the humoral immune response. In the meantime, confident identification of experienced individuals can inform evaluation of the impact of infection history and timing on the immunogenicity of candidate vaccines, and the experimental and analytical pipeline presented here could be deployed on vaccine trial samples to look for relationships between humoral immunity and infection or cCMV transmission risk.

Overall, many open questions regarding the role of humoral immunity in the context of CMV infection, transmission, latency, and reactivation remain. Higher resolution and more comprehensive analysis of antibody responses using systems serology approaches has the potential to improve our understanding of the complex virus-host interactions at play. Here, by analyzing highly curated cohorts, we report and validate phenotypic signatures of gB, pentamer, and tegument-specific antibody responses that not only robustly classify primary infection status, but also provide insights into time of infection. It remains to be seen if the atypical dynamic profile of IgG3 responses to envelope glycoproteins elicited by CMV is antigen-intrinsic and might be recapitulated when these antigens are delivered by other means, or if it may represent an evasion strategy dependent on other viral genes or aspects of the innate response to viral infection in the context of this notoriously immunoevasive virus. In the meantime, we believe that this work stands to define hallmarks of primary CMV infection and time of infection that may present new opportunities to streamline primary infection diagnosis. In particular, we hope that this will affect current clinical practice and enrolment of pregnant women with primary infection in interventional trials, thereby providing new insights into relative cCMV risk and management strategies.

## Methods

### Sex as a biological variable.

Our study examined the differences in individuals who were and were not pregnant following CMV infection. Pregnant individuals were female. Differences associated with biological sex among individuals who were not pregnant are presented in Supplemental Figure 4.

### Clinical samples.

Serum samples were gathered from participant cohort groups of pregnant primary infection, pregnant latent infection, nonpregnant primary infection, nonpregnant latent infection, as well as a CMV-negative patient cohort as a negative control group (Table 1). Human participants were recruited from Fondazione IRCCS Policlinico San Matteo, Pavia, Italy, and included participants who were healthy, primary, and chronically CMV infected, as well as those who were and were not pregnant. Diagnosis of primary CMV infection in the Pavia cohorts was based on 2 or more of the following criteria: CMV-specific IgG seroconversion, CMV-specific IgM antibody detection, or low IgG avidity index and CMV DNAemia. Chronic infection was defined by the presence of CMV-specific IgG, the absence of CMV-specific IgM, and no detection of CMV DNA in blood, saliva, urine, and genital secretions. Primary infection among participants who were not pregnant was diagnosed similarly with the exception that DNAemia was not assessed.

HCMV-specific IgG and IgM were determined by ETI-CYTOK-G and ETI-CYTOK-M (DiaSorin, Saluggia, Italy). IgM results obtained by the commercial assay were confirmed by an in-house developed capture ELISA assay (76). IgG avidity index was determined by an in-house developed ELISA test using HCMV nuclear antigen (59). The avidity index was defined as low if it was less than 35%, typically representing a primary infection acquired less than 12 weeks earlier, with avidity index values of less than 15% indicating an infection acquired less than 6 weeks earlier.

In 49 of 74 (66%) women who were pregnant and 26 of 27 (96%) participants who were not pregnant, time of onset of primary infection was defined by the appearance of symptoms, while in 19 of 74 (26%) of pregnant women who were asymptomatic, onset of infection was estimated on seroconversion (i.e. in the midpoint between the last IgG negative and the first IgG positive test result), occurring within a less than or equal to 6-week interval. Finally, in 6 (8%) asymptomatic women who were pregnant and 1 (3%) participant who was asymptomatic not pregnant, onset of infection was estimated on the kinetics of CMV-specific IgM and IgG avidity index. For a set of 40 pregnant women and 28 participants who were not pregnant with primary infection, 2–4 sequential serum samples collected until 6 months after onset of infection were available. Longitudinal samples from pregnant women with chronic infection were collected at 10, 20, and 30 weeks of gestation and at delivery. An additional cohort of pregnant women with primary infection was recruited from Erasme Hospital, as previously described (39, 77). Diagnosis of primary CMV infection was made by either documented IgG seroconversion, or increased titers of CMV-specific IgM at a subsequent sampling.

IgG seroconversion was not observed in all individuals, and not all participants were symptomatic. As a result, in some cases, primary infection status was inferred and time of infection was estimated from clinical assessments. Unsupervised analysis of data generated in this study indicated the lack of systematic bias associated with samples for which primary infection was diagnosed by inference compared with those for which IgG seroconversion was observed, lending confidence to these inferences (Supplemental Figure 8).

### Antibody profiling experiments.

Fc receptors were expressed as previously described (80). A complete list of Fc detection reagents and antigens used is provided in Supplemental Table 1. Pentamer and glycoprotein B were sourced both commercially, through industry partners, and expressed in-house. For the latter, HCMV Pentamer (UT, Towne strain NCBI taxonomy ID 10363) was produced by mixing plasmids encoding for residues 24 to 718 of gH with a C-terminal 6× HisTag, residues 31 to 278 of gL, residues 21 to 171 of UL128, residues 26 to 214 of UL130, and residues 19 to 129 of UL131A, all with artificial signal sequences, at an equimolar ratio (36). This mixture was then used to transiently transfect FreeStyle293-F cells via polyethylenimine. Plasmids encoding for postfusion HCMV gB (AD169 strain NCBI taxonomy ID 10360) residues 32–692 with an artificial N-terminal signal sequence and a C-terminal HRV3C protease cleavage site, 8×HisTag, and a TwinStrep tag. One postfusion gB construct (UT, or JSM-1074) contained substitutions Y155G, I156H, Y157R, Y206H, S238N, W240T, L241T, Y242H, and C246S, which have been reported previously (35), and the other construct (3p, or JSM-956) contained substitutions Y155G, I156H, Y157R, Y206H, W240A, L241T, Y242H, C246S, R456A, R459G, M472P, R491P, G492P, and a C-terminal T4 fibritin trimerization motif (Supplemental Figure 9). Plasmids were transiently transfected into FreeStyle 293-F cells via polyethylenimine. Pentamer was purified using NiNTA resin (Thermo Fisher Scientific) and postfusion gB proteins were purified using Strep-Tactin affinity resin (IBA Lifesciences). Affinity-purified proteins were further purified by size-exclusion chromatography using a Superose6 10/300 column (Cytiva) in a buffer composed of 2 mM Tris pH 8.0, 200 mM NaCl, 0.02% NaN3.

The tegument antigens tested were chosen based on prior studies of the pp150 (UL32) and pp52 (UL44) CMV proteins. CG1 included aa 495-691 and aa 862-1048 of pp150 whereas CG2 included aa 695-864 of pp150 and aa 297-433 of pp52. These 2 polypeptides were previously described as immunodominant targets for serodiagnosis of primary and chronic CMV infection (37, 38).

Characterization of antibody profiles was performed using the Fc array assay (79, 80). Antigens were covalently coupled to magnetic microspheres (Luminex Corporation) using carbodiimide chemistry. Serum dilutions used in assays ranged from 1:250–1:5,000 based on initial pilot experiments and previous experience (Supplemental Table 1). Detection of antigen specific antibodies was done using R-phycoerythrin–conjugated secondary reagents specific to human immunoglobulin isotypes and subclasses and by Fc receptor tetramers. Median fluorescent intensity (MFI) data was acquired on a FlexMap 3D array reader (Luminex Corporation). Samples from CMV-naive individuals were tested to establish the specificity of measurements, and MFI values were not quantitatively compared between antigen-specificities or detection reagents. Samples were tested in technical duplicates and results were averaged.

### Classification of CMV infection status.

A binomial logistic regression model with least absolute shrinkage and selection operator (LASSO) regularization was used to prediction infection status. Model training was performed using the scikit-learn (version 1.3) in Python (version 3.9) with default options. The regularization parameter was chosen using the option that gave the lowest classification error. The model was trained to minimize the log loss function and the class boundary was set at a probability value of 0.5. Model accuracy was determined by the test-set label predictions compared with true labels. Accuracy was assessed over 100 repetitions of 5-fold cross validation. Permutation testing was done to measure model robustness by performing the same procedure as described above but on data for which class labels had been randomly shuffled. Feature importance was determined from a final model that included all participants. While other model architectures were tested and resulted in similar prediction accuracies, logistic regression results were selected for presentation given their simplicity and interpretability.

### Prediction of time since infection.

The same machine learning model as described for predicting CMV infection status was used, this time minimizing mean squared error in time since infection. The model was trained on cross sectional sample data and tested on longitudinal and Erasme samples.

### Statistics.

Statistical analysis was performed in GraphPad Prism (version 9.7). UMAP plots were generated in Python (version 3.9) using the umap-learn package (version 0.4) (81) and then plotted using Prism. Volcano plots were generated in R (version 4.3) using ggplot2. Statistical tests are described in the relevant figure legends and include Mann-Whitney tests and calculation of Pearson and R2 correlation coefficients.

### Study approval.

The study was approved by the IRBs at Fondazione IRCCS Policlinico San Matteo and Erasme Hospital for sample collection, and Dartmouth College for sample testing and analysis. Each participant gave written informed consent.

### Data availability.

Data used in the study is available as Supporting data values file. Code is available at https://github.com/AckermanLab/Andrew_paper1/commit/22c6397e988356150e2c2c8836563e5e30428840

## Author contributions

Study conceptualization was contributed by APH, DL, AM, and MEA. Study methodology was contributed by APH, CALR, SS, HN, JAW, and DW. Software was developed by APH and CALR. Experimental investigation was conducted by APH, SS, HN, CD, MLD, PD, MF, and CF. Resources were provided by DW, JSM, DL, and AM. The original draft was written by APH and MEA. Supervision was provided by JSM, DL, AM, and MEA. Funding acquisition was provided by DL, AM, and MEA. All authors contributed to review and editing of the manuscript.

## Supplementary Material

Supplemental data

ICMJE disclosure forms

Supporting data values

## Figures and Tables

**Table 1:**
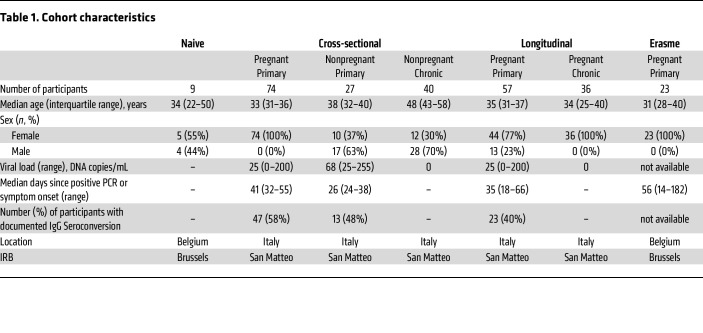
Cohort characteristics

## Version 1 08/29/2024

In-Press Preview

## Version 2 10/15/2024

Electronic publication

## References

[1] Cannon MJ, Davis KF (2005). Washing our hands of the congenital cytomegalovirus disease epidemic. BMC Public Health.

[2] Schleiss MR (2011). Congenital cytomegalovirus infection: molecular mechanisms mediating viral pathogenesis. Infect Disord Drug Targets.

[3] Dietrich ML, Schieffelin JS (2019). Congenital cytomegalovirus infection. Ochsner J.

[4] Pass RF, Anderson B (2014). Mother-to-child transmission of cytomegalovirus and prevention of congenital infection. J Pediatric Infect Dis Soc.

[5] Ross SA (2006). Hearing loss in children with congenital cytomegalovirus infection born to mothers with preexisting immunity. J Pediatr.

[6] Stagno S (1982). Congenital cytomegalovirus infection: The relative importance of primary and recurrent maternal infection. N Engl J Med.

[7] Lazzarotto T (2020). Congenital cytomegalovirus infection: a narrative review of the issues in screening and management from a panel of European experts. Front Pediatr.

[8] Simonazzi G (2018). Perinatal outcomes of non-primary maternal cytomegalovirus infection: a 15-year experience. Fetal Diagn Ther.

[9] Gatta LA (2022). Clinical factors associated with cytomegalovirus shedding among seropositive pregnant women. Am J Obstet Gynecol MFM.

[10] Zelini P (2022). Human cytomegalovirus non-primary infection during pregnancy: antibody response, risk factors and newborn outcome. Clin Microbiol Infect.

[11] Kenneson A, Cannon MJ (2007). Review and meta-analysis of the epidemiology of congenital cytomegalovirus (CMV) infection. Rev Med Virol.

[12] Revello MG (2014). A randomized trial of hyperimmune globulin to prevent congenital cytomegalovirus. N Engl J Med.

[13] Hughes BL (2021). A trial of hyperimmune globulin to prevent congenital cytomegalovirus infection. N Engl J Med.

[14] Kagan KO (2021). Outcome of pregnancies with recent primary cytomegalovirus infection in first trimester treated with hyperimmunoglobulin: observational study. Ultrasound Obstet Gynecol.

[15] Kagan KO (2019). Prevention of maternal-fetal transmission of cytomegalovirus after primary maternal infection in the first trimester by biweekly hyperimmunoglobulin administration. Ultrasound Obstet Gynecol.

[16] Kagan KO (2018). Antenatal treatment options for primary cytomegalovirus infections. Curr Opin Obstet Gynecol.

[17] Ross SA (2011). Overview of the diagnosis of cytomegalovirus infection. Infect Disord Drug Targets.

[18] American Academy of Pediatrics, Joint Committee on Infant Hearing (2007). Year 2007 position statement: principles and guidelines for early hearing detection and intervention programs. Pediatrics.

[19] Prince HE, Lapé-Nixon M (2014). Role of cytomegalovirus (CMV) IgG avidity testing in diagnosing primary CMV infection during pregnancy. Clin Vaccine Immunol.

[20] Saldan A (2017). Testing for cytomegalovirus in pregnancy. J Clin Microbiol.

[21] Hughes BL, Gyamfi-Bannerman C (2016). Diagnosis and antenatal management of congenital cytomegalovirus infection. Am J Obstet Gynecol.

[22] Abdullahi Nasir I (2016). Clinical significance of IgG avidity testing and other considerations in the diagnosis of congenital cytomegalovirus infection: a review update. Med Sci (Basel).

[23] Chatzakis C (2020). Timing of primary maternal cytomegalovirus infection and rates of vertical transmission and fetal consequences. Am J Obstet Gynecol.

[24] Aghaeepour N (2017). An immune clock of human pregnancy. Sci Immunol.

[25] Kourtis AP (2014). Pregnancy and infection. N Engl J Med.

[26] Abu-Raya B (2020). Maternal immunological adaptation during normal pregnancy. Front Immunol.

[27] Zuniga EI (2015). Innate and adaptive immune regulation during chronic viral infections. Annu Rev Virol.

[28] Rouse BT, Sehrawat S (2010). Immunity and immunopathology to viruses: what decides the outcome?. Nat Rev Immunol.

[29] Ovies C (2021). Pregnancy influences immune responses to SARS-CoV-2. Sci Transl Med.

[30] Steinhoff MC (2010). Influenza immunization in pregnancy--antibody responses in mothers and infants. N Engl J Med.

[31] Atyeo CG (2022). Maternal immune response and placental antibody transfer after COVID-19 vaccination across trimester and platforms. Nat Commun.

[32] Ripalti A (1994). Construction of polyepitope fusion antigens of human cytomegalovirus ppUL32: reactivity with human antibodies. J Clin Microbiol.

[33] Chandramouli S (2015). Structure of HCMV glycoprotein B in the postfusion conformation bound to a neutralizing human antibody. Nat Commun.

[34] Chandramouli S (2017). Structural basis for potent antibody-mediated neutralization of human cytomegalovirus. Sci Immunol.

[35] Ye X (2020). Recognition of a highly conserved glycoprotein B epitope by a bivalent antibody neutralizing HCMV at a post-attachment step. PLoS Pathog.

[36] Wrapp D (2022). Structural basis for HCMV pentamer recognition by neuropilin 2 and neutralizing antibodies. Sci Adv.

[37] Vornhagen R (1996). Immunoglobulin A-specific serodiagnosis of acute human cytomegalovirus infection by using recombinant viral antigens. J Clin Microbiol.

[38] Vornhagen R (1994). Early serodiagnosis of acute human cytomegalovirus infection by enzyme-linked immunosorbent assay using recombinant antigens. J Clin Microbiol.

[39] Antoine P (2012). Functional exhaustion of CD4+ T lymphocytes during primary cytomegalovirus infection. J Immunol.

[40] Rozsnyay Z (1989). Distinctive role of IgG1 and IgG3 isotypes in Fc gamma R-mediated functions. Immunology.

[41] Bruhns P (2009). Specificity and affinity of human Fcgamma receptors and their polymorphic variants for human IgG subclasses. Blood.

[42] Nachbagauer R (2016). Hidden Staphylococcus aureus carriage: overrated or underappreciated?. mBio.

[43] Yates NL (2011). Multiple HIV-1-specific IgG3 responses decline during acute HIV-1: implications for detection of incident HIV infection. AIDS.

[44] Doerr HW (1987). Serologic detection of active infections with human herpes viruses (CMV, EBV, HSV, VZV): diagnostic potential of IgA class and IgG subclass-specific antibodies. Infection.

[45] Gilljam G, Wahren B (1989). Properties of IgG subclasses to human cytomegalovirus. J Virol Methods.

[46] Gupta CK (1996). IgG subclass antibodies to human cytomegalovirus (CMV) in normal human plasma samples and immune globulins and their neutralizing activities. Biologicals.

[47] Joassin L (1989). Detection by enzyme-linked immunosorbent assay of specific immunoglobulin G isotypes in primary and established cytomegalovirus infections. J Clin Microbiol.

[48] Rodriguez GE, Adler SP (1990). Immunoglobulin G subclass responses to cytomegalovirus in seropositive patients after transfusion. Transfusion.

[49] Atalay R (2002). Identification and expression of human cytomegalovirus transcription units coding for two distinct Fcgamma receptor homologs. J Virol.

[50] Corrales-Aguilar E (2014). CMV-encoded Fcγ receptors: modulators at the interface of innate and adaptive immunity. Semin Immunopathol.

[51] Cortese M (2012). Recombinant human cytomegalovirus (HCMV) RL13 binds human immunoglobulin G Fc. PLoS One.

[52] Sprague ER (2008). The human cytomegalovirus Fc receptor gp68 binds the Fc CH2-CH3 interface of immunoglobulin G. J Virol.

[53] Powers C (2008). Cytomegalovirus immune evasion. Curr Top Microbiol Immunol.

[54] Jackson SE (2017). CMV immune evasion and manipulation of the immune system with aging. Geroscience.

[55] Genser B (2001). Evaluation of five commercial enzyme immunoassays for the detection of human cytomegalovirus-specific IgM antibodies in the absence of a commercially available gold standard. Clin Chem Lab Med.

[56] Rasmussen L (1982). Virus-specific IgG and IgM antibodies in normal and immunocompromised subjects infected with cytomegalovirus. J Infect Dis.

[57] Naumnik B (2007). Comparison of serology assays and polymerase chain reaction for the monitoring of active cytomegalovirus infection in renal transplant recipients. Transplant Proc.

[58] Razonable RR (2020). Clinical diagnostic testing for human cytomegalovirus infections. J Infect Dis.

[59] Revello MG (2010). Comparative evaluation of eight commercial human cytomegalovirus IgG avidity assays. J Clin Virol.

[60] Dugast A-S (2014). Independent evolution of Fc- and Fab-mediated HIV-1-specific antiviral antibody activity following acute infection. Eur J Immunol.

[61] Sadanand S (2018). Temporal variation in HIV-specific IgG subclass antibodies during acute infection differentiates spontaneous controllers from chronic progressors. AIDS.

[62] Damelang T (2019). Role of IgG3 in infectious diseases. Trends Immunol.

[63] Dauby N (2016). Limited effector memory B-cell response to envelope glycoprotein B during primary human cytomegalovirus infection. J Infect Dis.

[64] Corrales-Aguilar E (2014). Human cytomegalovirus Fcγ binding proteins gp34 and gp68 antagonize Fcγ receptors I, II and III. PLoS Pathog.

[65] Antonsson A, Johansson PJH (2001). Binding of human and animal immunoglobulins to the IgG Fc receptor induced by human cytomegalovirus. J Gen Virol.

[66] Vezzani G (2022). Human immunoglobulins are transported to HCMV viral envelope by viral Fc gamma receptors-dependent and independent mechanisms. Front Microbiol.

[67] Ssentongo P (2021). Congenital cytomegalovirus infection burden and epidemiologic risk factors in countries with universal screening: a systematic review and meta-analysis. JAMA Netw Open.

[68] Wang C (2011). Attribution of congenital cytomegalovirus infection to primary versus non-primary maternal infection. Clin Infect Dis.

[69] Picone O (2013). A series of 238 cytomegalovirus primary infections during pregnancy: description and outcome. Prenat Diagn.

[70] Lazzarotto T (2011). Update on the prevention, diagnosis and management of cytomegalovirus infection during pregnancy. Clin Microbiol Infect.

[71] Nigro G (2005). Passive immunization during pregnancy for congenital cytomegalovirus infection. N Engl J Med.

[72] Jückstock J (2015). Passive Immunization against congenital cytomegalovirus infection: current state of knowledge. Pharmacology.

[73] Gong Y (2023). Mathematical modeling of rhesus cytomegalovirus transplacental transmission in seronegative rhesus macaques. Viruses.

[74] Otero CE (2023). Relationship of maternal cytomegalovirus-specific antibody responses and viral load to vertical transmission risk following primary maternal infection in a rhesus macaque model. PLoS Pathog.

[75] Semmes EC (2023). ADCC-activating antibodies correlate with decreased risk of congenital human cytomegalovirus transmission. JCI Insight.

[76] Grazia Revello M (1991). Development and evaluation of a capture ELISA for IgM antibody to the human cytomegalovirus major DNA binding protein. J Virol Methods.

[77] Huygens A (2015). Functional exhaustion limits CD4+ and CD8+ T-cell responses to congenital cytomegalovirus infection. J Infect Dis.

[78] Boesch AW (2014). Highly parallel characterization of IgG Fc binding interactions. MAbs.

[79] Brown EP (2017). Multiplexed Fc array for evaluation of antigen-specific antibody effector profiles. J Immunol Methods.

[80] Brown EP (2018). Optimization and qualification of an Fc array assay for assessments of antibodies against HIV-1/SIV. J Immunol Methods.

