# Supplementary material for: Discrimination of primary and chronic cytomegalovirus infection based on humoral immune profiles in pregnancy
Source: J Clin Invest. 2024 Oct 15;134(20):e180560. doi: 10.1172/JCI180560 (PMC11473158; doi:10.1172/JCI180560)
Supplement: Supplemental data [file jci-134-180560-s019.pdf]

Supplementary Materials

**Supplementary Figures**	
Supplementary Figure 1	Heatmap of antibody responses.
Supplementary Figure 2	Comparison of antibody responses in pregnant and non-pregnant female individuals.
Supplementary Figure 3	Comparison of antibody responses in pregnant individuals from Erasme and those from other medical centers.
Supplementary Figure 4	Comparison of male and female antibody responses in non-pregnant subjects following primary or chronic CMV infection
Supplementary Figure 5	Representative boxplots of CMV-specific antibody binding to FcγR across groups.
Supplementary Figure 6	Longitudinal CMV cohort UMAP
Supplementary Figure 7	Longitudinal machine learning model performance and feature importance
Supplementary Figure 8	UMAP of subjects with primary CMV infection by IgG seroconversion status.
Supplementary Figure 9	3p gB and UT gB construct design
**Supplementary Tables**	
Supplementary Table 1	Fc detection and antigen reagents.

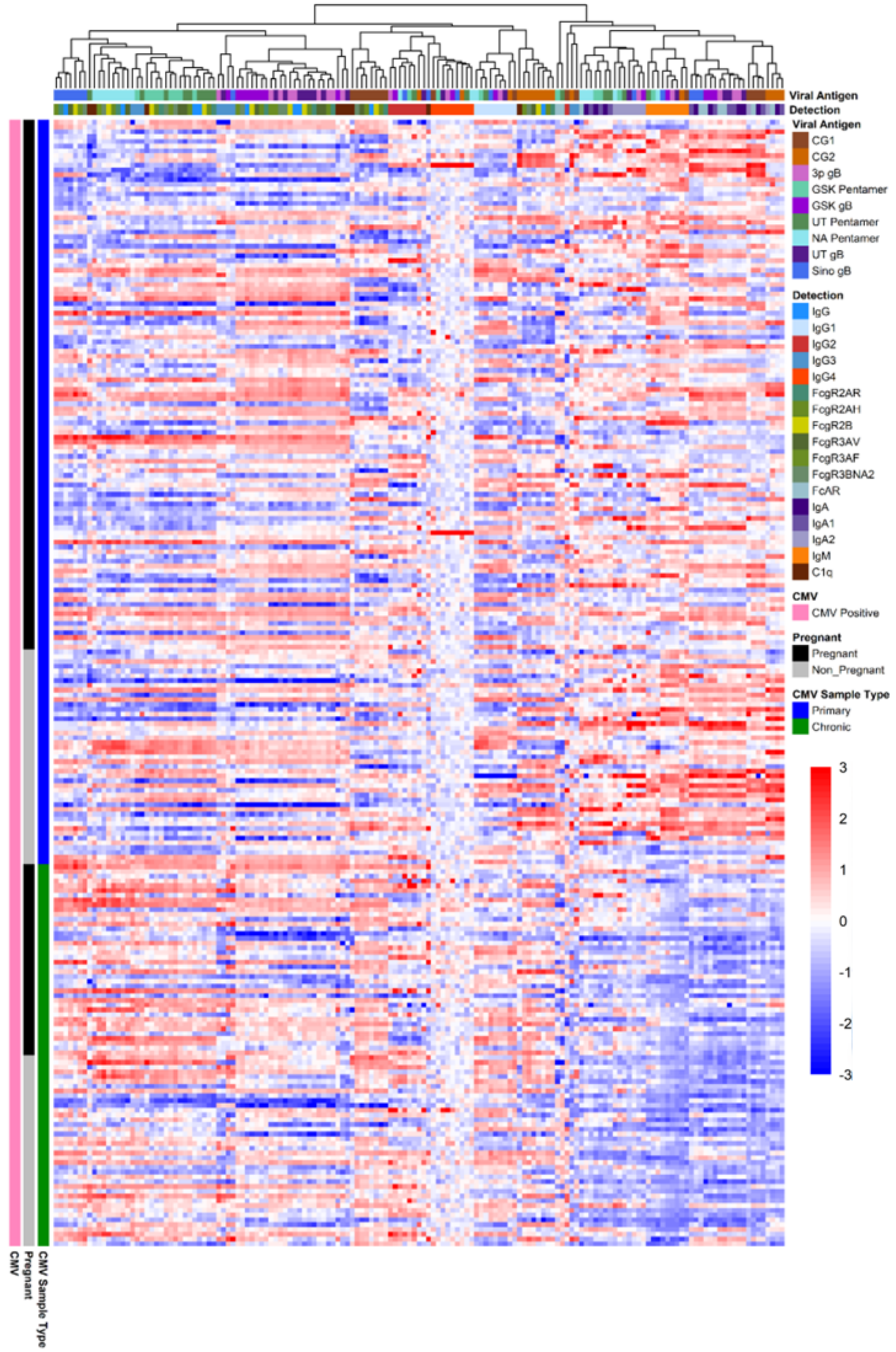

Supplemental Figure 1: Heatmap of hierarchically clustered CMV-specific Fc array features across subjects in the cross-sectional cohort. Each row represents an individual subject. Subjects are grouped by status, as indicated by the vertical color bars. Each column represents an Fc array feature; horizontal color bars indicate each function or each Fv-specificity (Antigen) and Fc-characteristic (Detection) tested. Responses are scaled and centered per feature and the range was truncated ± 3 SD.

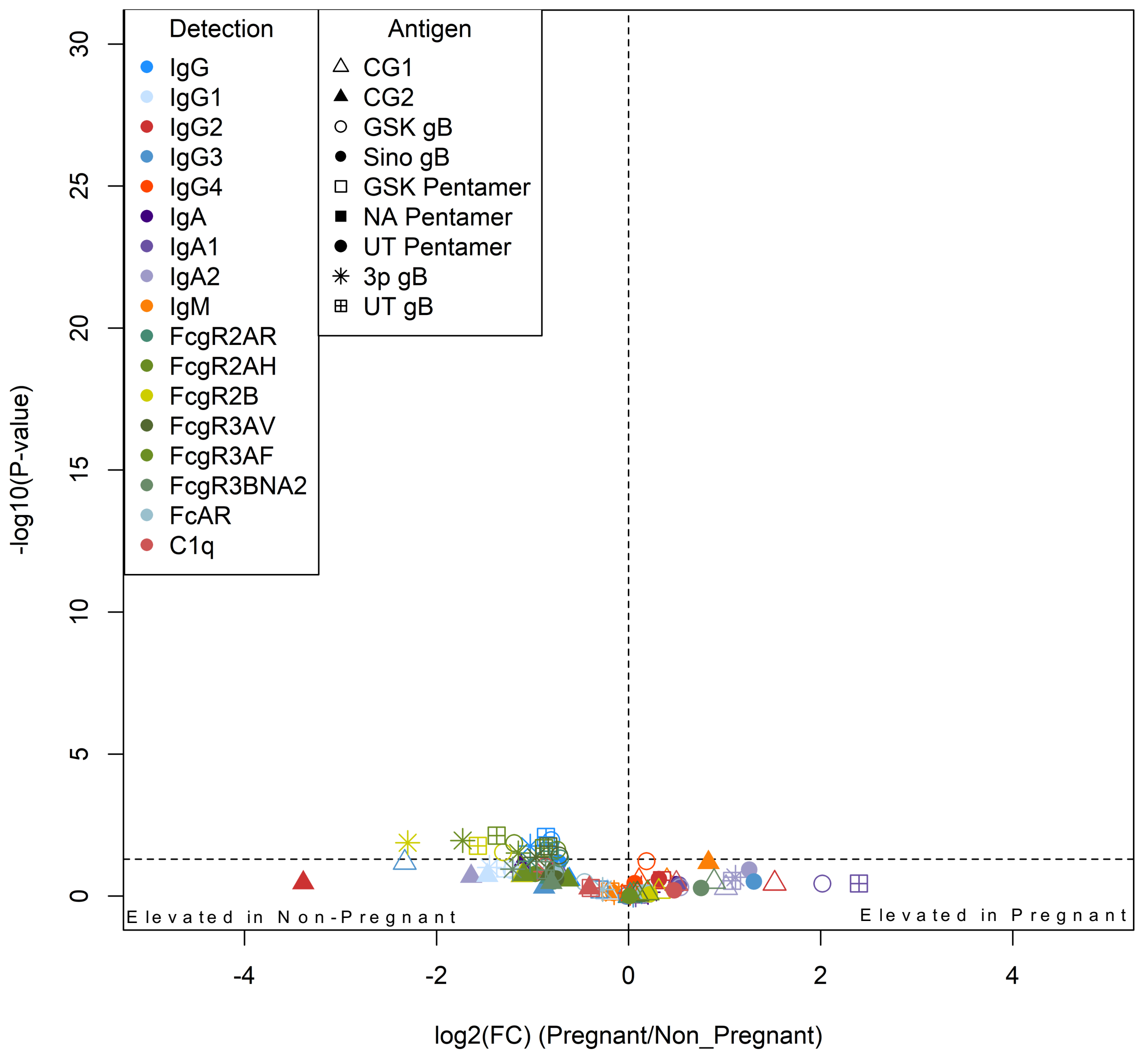

Supplemental Figure 2: Comparison of pregnant and non-pregnant antibody responses in female subjects. Volcano plot of each CMV-specific antibody feature assessed for pregnant female (n=118) and non-pregnant female (n=24) subjects. Volcano plot represents the log2 fold change (x-axis) against the –log10 p value (Mann Whitney test). Antibody specificities (Antigen) are indicated by shape and Fc characteristics (Detection) indicated by color.

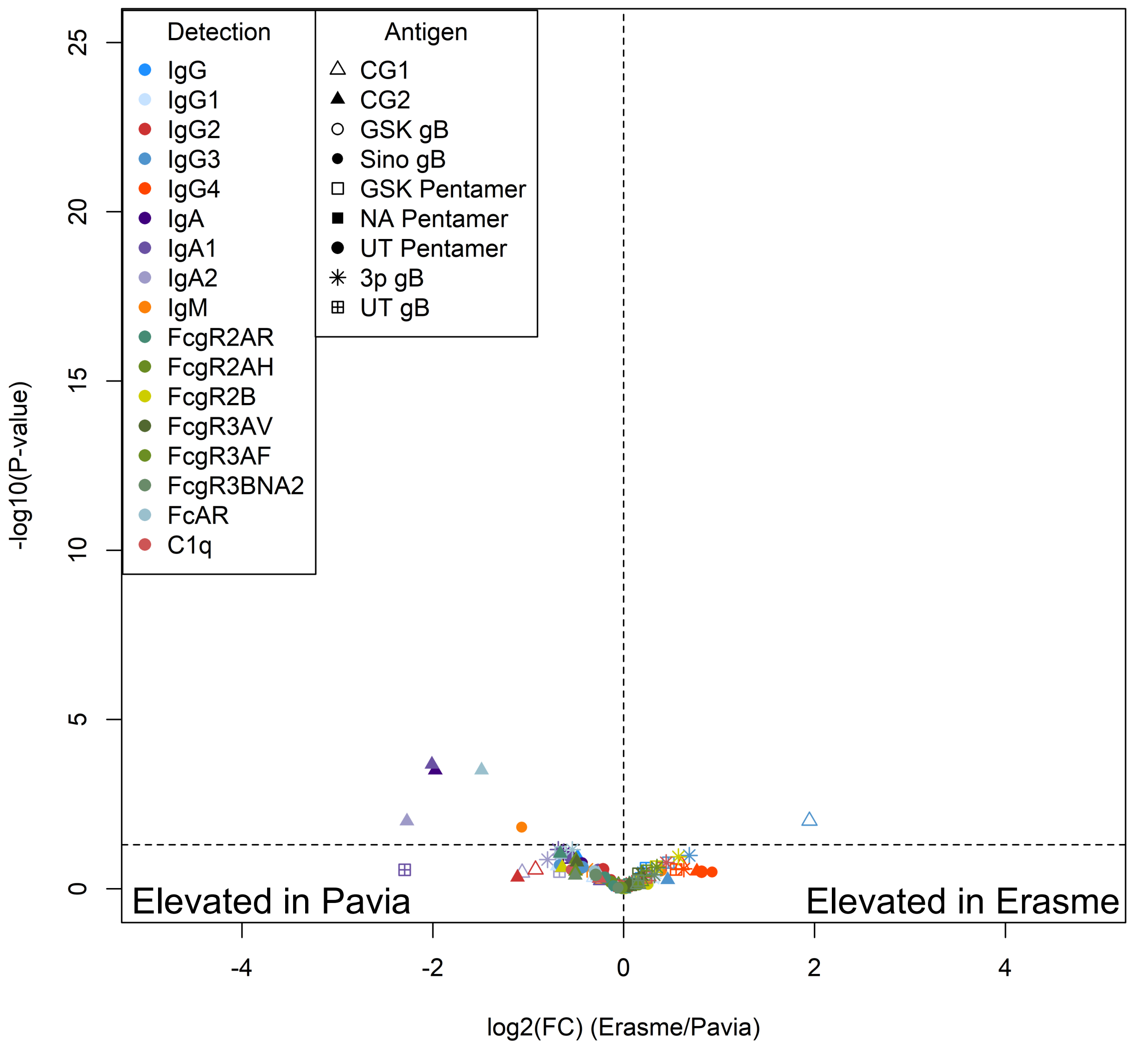

Supplemental Figure 3. Comparison of antibody responses across medical centers. Volcano plot of each CMV-specific antibody feature assessed. Volcano plot represents the log2 fold change (x-axis) against the –log10 p value (Mann Whitney test). Antibody specificities (Antigen) are indicated by shape and Fc characteristics (Detection) indicated by color.

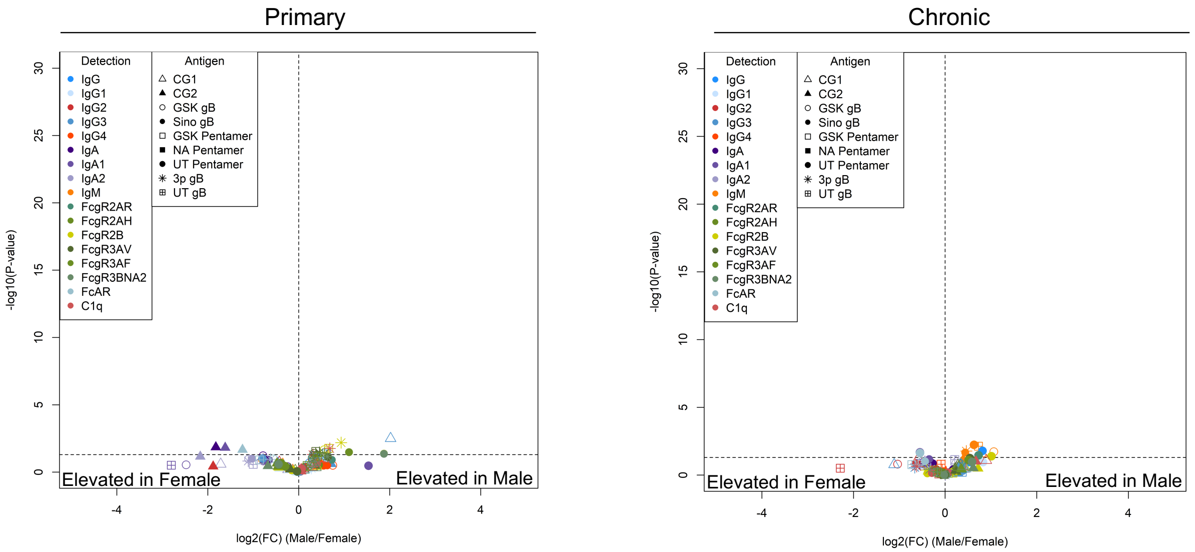

Supplemental Figure 4. Comparison of male and female antibody responses in non-pregnant subjects following primary or chronic CMV infection. Volcano plot of each CMV-specific antibody feature assessed in primary (left) CMV subjects reported as male (n=10) versus female (n=17) and chronic (right) CMV subjects reported as male (n=12) versus female (n=28). Volcano plot represents the log2 fold change (x-axis) against the –log10 p value (Mann Whitney test). Antibody specificities (Antigen) are indicated by shape and Fc characteristics (Detection) indicated by color.

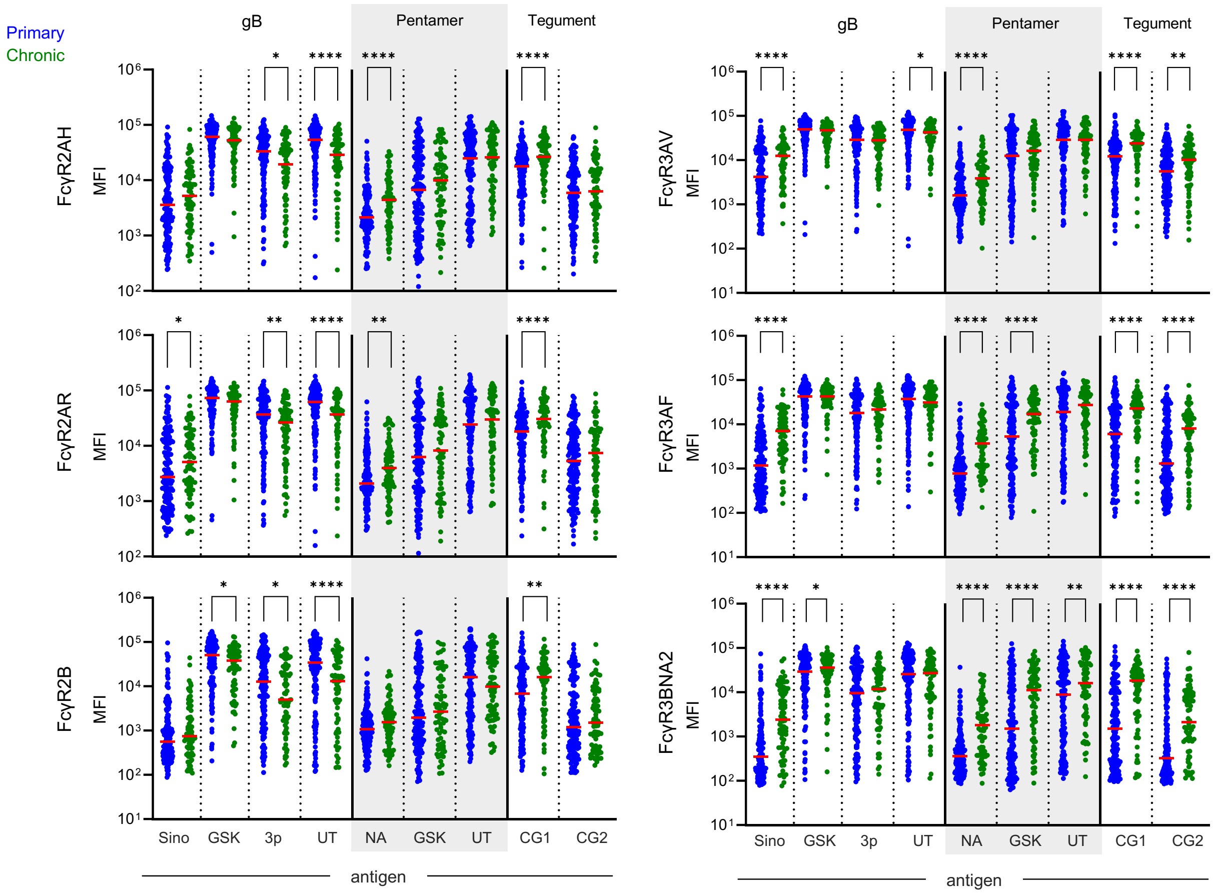

Supplemental Figure 5. Representative boxplots of CMV-specific antibody binding to FcγR across groups. FcγR2AH, FcγR2AR, FcγR2B, FcγR3AV, FcγR3AF, FcγR3BNA2 levels, as defined by median fluorescent intensity (MFI) in subjects with primary (blue) or chronic (green) CMV infection. Values represent the mean of technical replicates; bars indicate group medians (Mann-Whitney test: *p < 0.05, **p < 0.01, ***p < 0.001, and ****p < 0.0001).

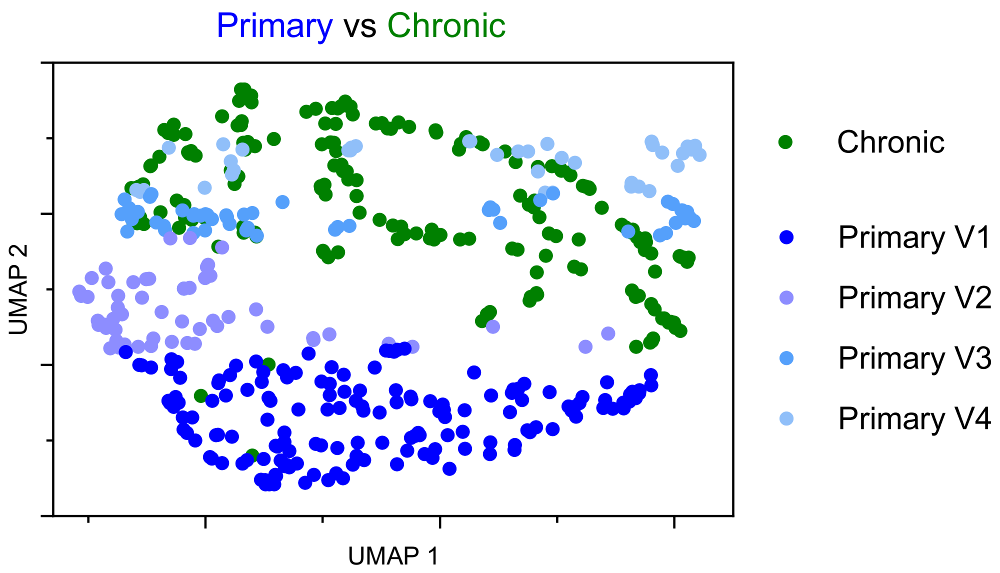

Supplemental Figure 6. CMV Longitudinal Cohort UMAP. UMAP of longitudinal primary and chronic CMV infection samples projected onto the UMAP dimensional space defined by visit 1 samples. Uniform manifold approximation (UMAP) biplot of antibody feature profile for subjects with primary CMV infection colored by visit number (blue) and chronic CMV infected subjects (green).

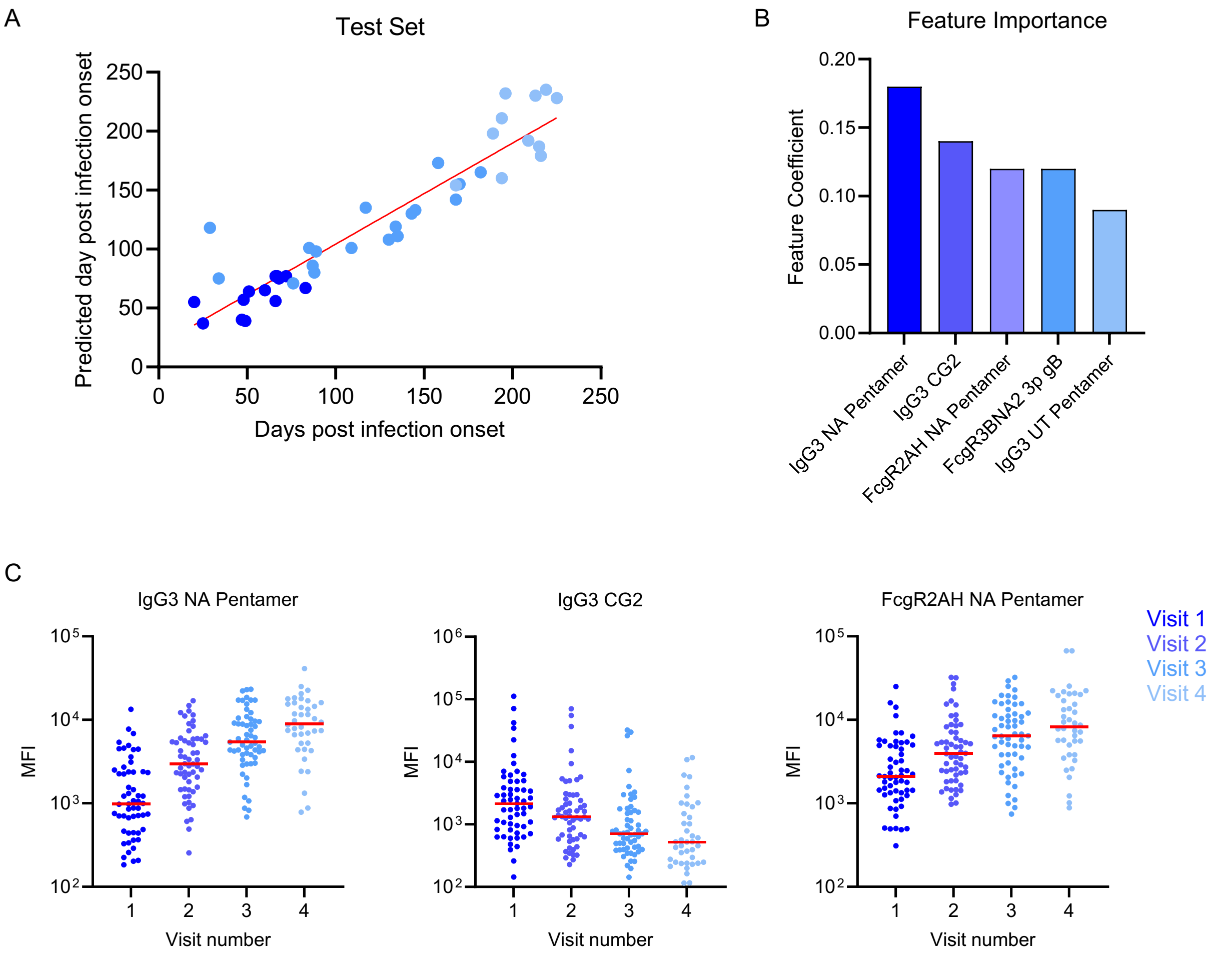

Supplemental Figure 7. Longitudinal machine learning model performance and feature importance. A. Representative test set for predicting days post infection. Symbols are colored according to visit number. Red line denotes the best fit line x=y. **B.** Top five model features according to feature importance. **C.** Boxplots of the top three model features plotted according to visit number.

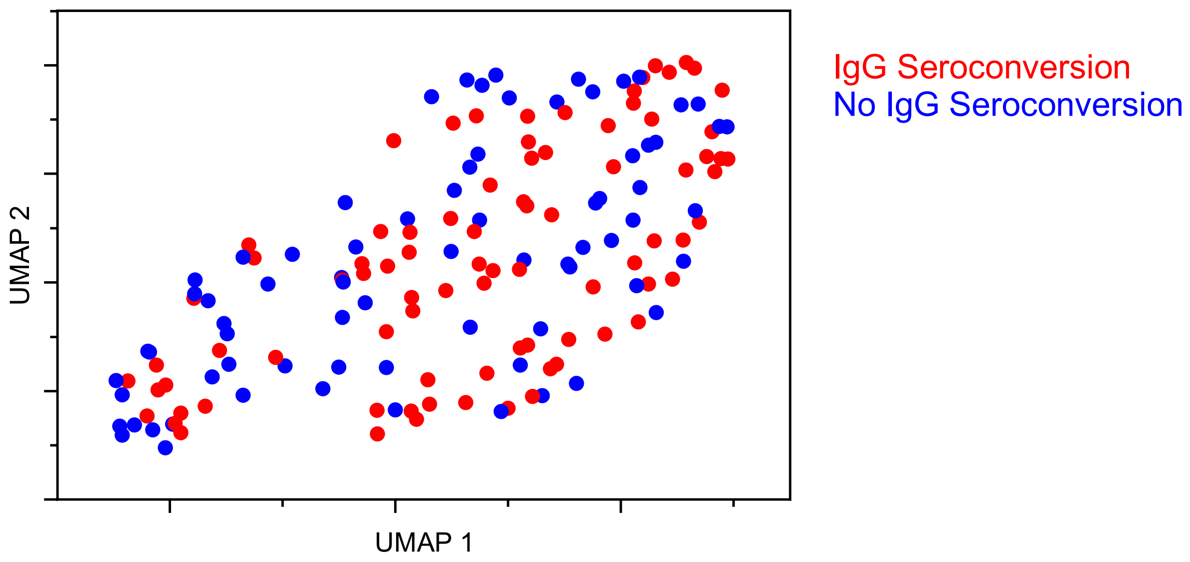

Supplemental Figure 8. UMAP of subjects with primary CMV infection by IgG seroconversion status. Uniform manifold approximation (UMAP) biplot of antibody feature profile for subjects with primary CMV infection seroconversion was (red) or was not (blue) observed during sample collection.

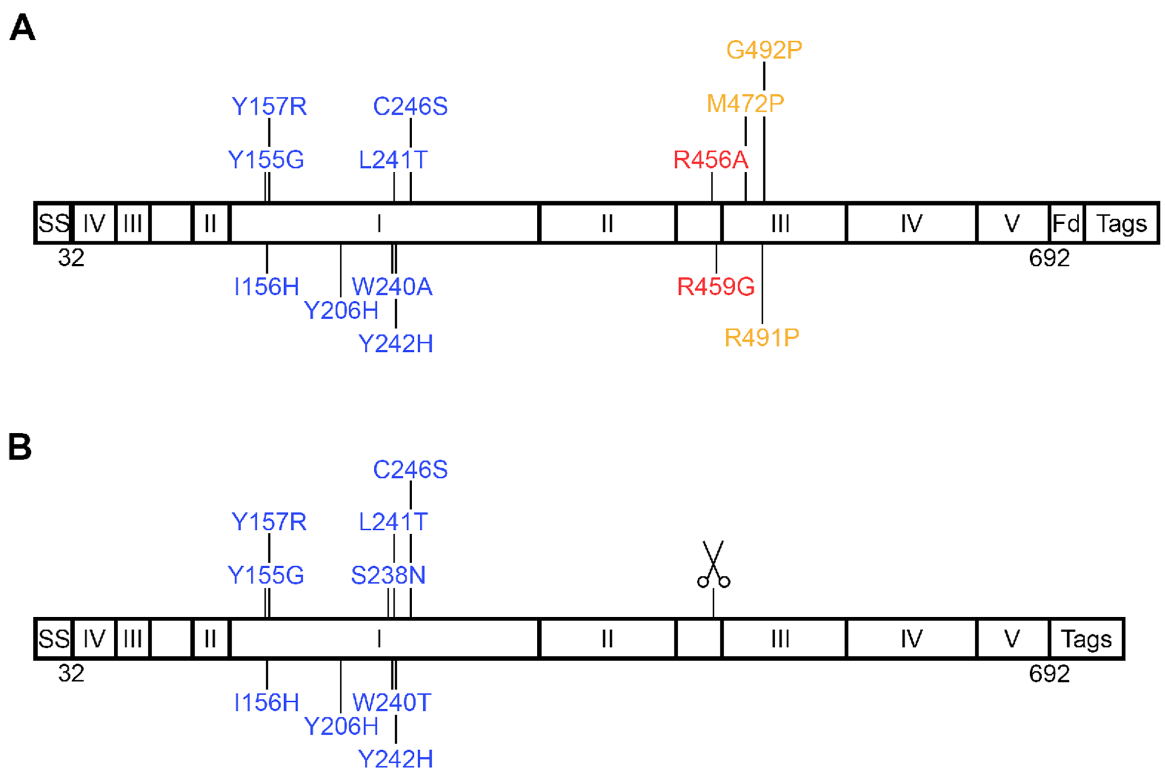

Supplemental Figure 9. Sequence alignment of 3p gB and UT gB constructs.
A. Sequence schematic of “JSM-956” (3p gB) and **B.** sequence schematic of “JSM-1074” (UT gB). Mutations intended to enhance solubility are colored blue, mutations to disrupt furin cleavage are colored red and proline mutations are colored yellow. SS=signal sequence, I=domain I, II=domain II, III=domain III, IV=domain IV, V=domain V, Fd=foldon, and the scissors denote the native furin cleavage site.

Supplemental Table 1. Fc detection and antigen reagents

Antigen	Source	Strain/further details
**Other**		
Tetanus	Sigma 676570-37-9	
RSV dsCav1	McLellan et al., Science. 2013	
Pertactin	VWR 102946-462	
Rubella capsid	Abcam ab43034	
Neuraminidase	Immune Technology IT-003-00110p	
Hepatitis B	Zageno H1909-17C	
**CMV**		
gB UT (JSM-1074)	Ye et al., PLoS Pathog. 2020	Towne (NCBI taxonomy ID 10363)
gB 3p (JSM-956)	This study	Towne (NCBI taxonomy ID 10363)
gB GSK	Chandramouli et al., Nat Commun. 2015	Merlin
gB Sino	Sino Biological 10202-V08H1	Towne (GenBank: AAA45920.1)
Pentamer UT	Wrapp et al., Sci Adv. 2022	AD169 (NCBI taxonomy ID 10360)
Pentamer NA	Native Antigen CMV-PENT	VR1814 (NCBI Accession Code ACZ79986)
Pentamer GSK	Chandramouli et al., Sci Immunol. 2017	Merlin
Tegument CG1 (pp150/2-pp52/3)	Nexelis, Vornhagen et al., J Clin Microbiol. 1994	AD169; aa 695-864 of pp150 and aa 297-433 of pp52
Tegument CG2 (pp150/7-pp150/1)	Nexelis, Vornhagen et al., J Clin Microbiol. 1994	AD169; aa 495-691 and 862-1048 of pp150
**Fc Detection**	**Source**	**Serum dilution tested**
a- IgG	Southern Biotech 1030-09	1:5000
a-IgG1	Southern Biotech 9054-09	1:1000
a-IgG2	Southern Biotech 9070-09	1:250
a-IgG3	Southern Biotech 9210-09	1:250
a-IgG4	Southern Biotech 9200-09	1:250
a-IgA	Southern Biotech 2050-09	1:250
a-IgA1	Southern Biotech 9130-09	1:250
a-IgA2	Southern Biotech 9140-09	1:250
a-IgM	Southern Biotech 9020-09	1:250
FcγRIIa R131	Boesch et al., mAbs. 2014	1:5000
FcγRIIa H131	Boesch et al., mAbs. 2014	1:5000
FcγRIIb	Boesch et al., mAbs. 2014	1:5000
FcγRIIIa V158	Boesch et al., mAbs. 2014	1:5000
FcγRIIIa F158	Boesch et al., mAbs. 2014	1:5000
FcγRIIIb NA2	Boesch et al., mAbs. 2014	1:5000
FcαR	Duke Protein Production Facility	1:250
